# Effects of Bee Venom on Glutamate-Induced Toxicity in Neuronal and Glial Cells

**DOI:** 10.1155/2012/368196

**Published:** 2011-08-28

**Authors:** Sang Min Lee, Eun Jin Yang, Sun-Mi Choi, Seon Hwy Kim, Myung Gi Baek, Jing Hua Jiang

**Affiliations:** Department of Standard Research, Korea Institute of Oriental Medicine, 483 Expo-ro, Yuseong-gu, Daejeon, 305-811, Republic of Korea

## Abstract

Bee venom (BV), which is extracted from honeybees, is used in traditional Korean medical therapy. Several groups have demonstrated the anti-inflammatory effects of BV in osteoarthritis both *in vivo* and *in vitro*. Glutamate is the predominant excitatory neurotransmitter in the central nervous system (CNS). Changes in glutamate release and uptake due to alterations in the activity of glutamate transporters have been reported in many neurodegenerative diseases, including Parkinson's disease, Alzheimer's disease, and amyotrophic lateral sclerosis. To assess if BV can prevent glutamate-mediated neurotoxicity, we examined cell viability and signal transduction in glutamate-treated neuronal and microglial cells in the presence and absence of BV. We induced glutamatergic toxicity in neuronal cells and microglial cells and found that BV protected against cell death. Furthermore, BV significantly inhibited the cellular toxicity of glutamate, and pretreatment with BV altered MAP kinase activation (e.g., JNK, ERK, and p38) following exposure to glutamate. These findings suggest that treatment with BV may be helpful in reducing glutamatergic cell toxicity in neurodegenerative diseases.

## 1. Introduction

Glutamate is well known as the main excitatory neurotransmitter in the central nervous system (CNS), where it plays an important role in brain functions including memory, synaptic plasticity, learning, and cognition. Excitotoxicity is caused by the excessive and dysregulated activation of glutamate receptors [1]. Overstimulation of NMDA receptors by glutamate may underlie the abnormal neuronal degeneration observed following hypoxia, ischemia, hypoglycemia, and seizures [2].

Glutamate neurotoxicity is known to be associated with numerous neurodegenerative disorders, including Alzheimer's disease (AD), and it is considered to be a key factor in the pathogenesis of the disease [3]. Some studies have shown that amyloid-*β* protein (A*β*) inhibits glutamate uptake and leads to an increase in extracellular levels of glutamate [4]. It has also been reported that A*β* enhances glutamate-induced by excitotoxicity [5], suggesting that the neurotransmitter might mediate A*β*-induced cytotoxicity. *In vitro*, glutamate induces different types of cell death, including apoptosis and necrosis [6]. Recently, microglia have been linked to glutamate metabolism. Activated microglia from mice or macaques have been shown to express the excitatory amino acid transporter genes EAAT-1 and -2, suggesting that, similar to astrocytes, microglia take up glutamate and metabolize it to glutamine. Gras and colleagues have suggested that the clearance of extracellular glutamate and the synthesis of glutamine contribute to the neuroprotective properties of microglia in HIV-infected individuals [7].

In fact, motor neuron death may be mediated by numerous toxic factors that originate from different cell types. Among these factors, a role for the dysregulation of glutamate homeostasis in amyotrophic lateral sclerosis- (ALS)-mediated neurodegeneration has been established. In ALS patients, increased plasma levels of glutamate [8, 9], decreased glutamate uptake, decreased expression levels of the glial glutamate transporter EAAT2 [10, 11], and altered levels of glutamine synthetase [12] have been documented.

Bee venom (BV) is a traditional Korean medicine that has been successfully used to treat immune-related diseases, especially rheumatoid arthritis [13]. BV contains several biologically active peptides, including melittin, apamin, adolapin, and mast cell degranulating peptide [4]. Recent reports have indicated that acupuncture and the administration of BV can impart a significant antiarthritic response that is mediated by the inhibition of inflammatory mediators, similar to nonsteroidal anti-inflammatory drugs [14–16]. Jang and colleagues have shown that BV has anti-inflammatory effects on the Raw264.7 macrophage cell line that can be ascribed to the transcriptional downregulation of **inducible nitric oxide synthase** (iNOS), Cyclooxygenase-2 (COX-2), nuclear factor kappa B (NF-*κ*B) and mitogen-activated protein kinases (MAPKs) [17], and an antibacterial effect that has no side effects in animal models [18].

In this study, we report that pretreatment of neuronal and microglial cells with BV significantly inhibited glutamate-mediated toxicity. The glutamate-mediated toxicity required the c-Jun N-terminal kinase (JNK), extracellular signal-regulated kinase (ERK), p38, and alpha serine/threonine-protein kinase 1 (AKT) signaling pathways. In addition, pretreatment with BV significantly inhibited the expression of JNK, ERK, and p38 in glutamate-stimulated N2a neuronal cells.

The present results may have clinical implications and suggest that BV may be a potential treatment for the prevention of inflammatory or neurodegenerative diseases such as ALS.

## 2. Materials and Methods

### 2.1. Materials

BV, Tween-20, 3-(4,5-dimethylthiazol-2-yl)-2,5-diphenyltetrazolium bromide (MTT), and L-glutamate were purchased from Sigma (St. Louis, Mo, USA). Fetal bovine serum was purchased from Gibco (Grand Island, NY, USA). The lactate dehydrogenase (LDH) kit was purchased from Biovision Research Products (Biovision, Calif, USA). 

Primary antibodies specific for p-JNK, JNK, AKT, p-ERK, ERK, p-p38, and p38 were obtained from Cell Signaling Technology (San Francisco, Calif, USA). The primary antibody against p-AKT was obtained from Epitomics (Burlingame, Calif, USA).

### 2.2. Cell Culture

The N2a neuroblastoma cell line was purchased from ATCC (Manassas, Va, USA) and grown in Dulbecco's modified Eagle's medium (DMEM) supplemented with 10% FBS, 100 U/mL penicillin, and 100 *μ*g/mL streptomycin at 37°C in an atmosphere of 5% CO_2_ in air, as recommended by the ATCC. Cells were subcultured to a fresh culture dish when growth reached 70–90% confluence (i.e., every 2-3 days) as recommended by ATCC. For all experiments, proliferation state N2a neuroblastoma cells of passage number 20 or below were used.

The immortalized BV2 murine microglial cell line was provided by Dr. Sang-Myun Park (Aju University, Republic Korea) and cultured as described above.

In all experiments, cells were incubated in the presence or absence of the indicated concentrations of BV before the addition of glutamate to the culture media.

### 2.3. 3-(4,5-Dimethylthiazol-2-yl)-2,5-diphenyltetrazolium Bromide (MTT) Assay

This assay is based on the ability of active mitochondrial dehydrogenase to convert dissolved MTT into water-insoluble purple formazan crystals. Neuronal and microglial cells were plated in 96-well plates (1 × 10^4^ cells/well). After 24 h, the cells were treated with the indicated concentration of BV for 30 min prior to glutamate treatment for 24 h or 72 h. The MTT assay was performed as previously described. Briefly, MTT was added to each well at a final concentration of 0.5 mg/mL, and the plates were incubated for 1 h at 37°C. The culture medium was then removed, DMSO was added, and the plates were shaken for 10 min to solubilize the formazan reaction product. The absorbance at 570 nm was measured using a microplate reader (xMark, Bio-rad).

### 2.4. Determination of Membrane Integrity Using an LDH Release Assay

Cell membrane damage/cytotoxicity was determined using an LDH release assay, which quantitatively measures the activity of LDH, a stable cytosolic enzyme that is released upon cell membrane damage or cell lysis. For this experiment, neuronal and microglial cells were plated in 96-well plates (1 × 10^4^ cells/well). The cells were treated with the indicated concentration of BV for 30 min prior to glutamate treatment for 48 h. Culture supernatants were then collected from each well and assayed for LDH release according to the manufacturer's instructions. The absorbance at 450 nm following a 10 min reaction was measured by a microplate reader. The mean change in absorbance in culture supernatants was calculated for each treatment and is expressed as the percentage of the absorbance of control cell supernatants.

### 2.5. Western Blot Analysis

Following treatment, total cellular proteins were isolated using ice-cold lysis buffer containing 50 mM Tris HCl pH 7.4, 1% NP-40, 0.1% SDS, 150 mM NaCl, and the Complete Mini Protease Inhibitor Cocktail (Roche, Basel, Switzerland). The protein concentration was determined using a BCA Protein Assay Kit (Pierce, Ill, USA). Extracted samples (20 *μ*g total protein per lane) were separated using SDS-polyacrylamide gel electrophoresis (SDS-PAGE) and then transferred to nitrocellulose membranes (Whatman, Kan, USA). The membrane was then incubated with blocking solution (5% skim milk) to block nonspecific protein binding, followed by incubation with the following primary antibodies: phospho-Akt (pSer473), Akt, phospho-JNK, JNK, phospho-ERK, ERK, phospho-p38, p38, and *α*-tubulin (Abcam, Mass, USA). The membrane was incubated with a horseradish peroxidase- (HRP)-conjugated secondary antibody for 2 h at room temperature, and specific protein bands were detected using the SuperSignal West Femto Chemiluminescent Substrate (Pierce, Ill, USA) and enhanced chemiluminescence reagents (Amersham Pharmacia, NJ, USA). *α*-tubulin was used as an internal control to normalize protein loading. Protein bands were detected and analyzed using an LAS-3000 image analyzer (Fujifilm, Tokyo, Japan). Quantification of the blotting bands was performed using the Multi Gauge V3.0 software program (Fujifilm).

### 2.6. Statistical Analysis

Results are expressed as mean ± SEM values. Statistical evaluations were conducted using the *t*-test for comparisons immunoblotting between control and glutamate or BV-treated group. Graphpad Prism version 5 software program was used for all analyses. A value of *P* < 0.05 was considered significant. 

Differences in the MTT cell viability and LDH assays were analyzed with two-way ANOVA tests.

## 3. Results

### 3.1. Pretreatment with BV Protects against Glutamate-Induced Cell Death in N2a Neuronal Cells

**Figure 1:**
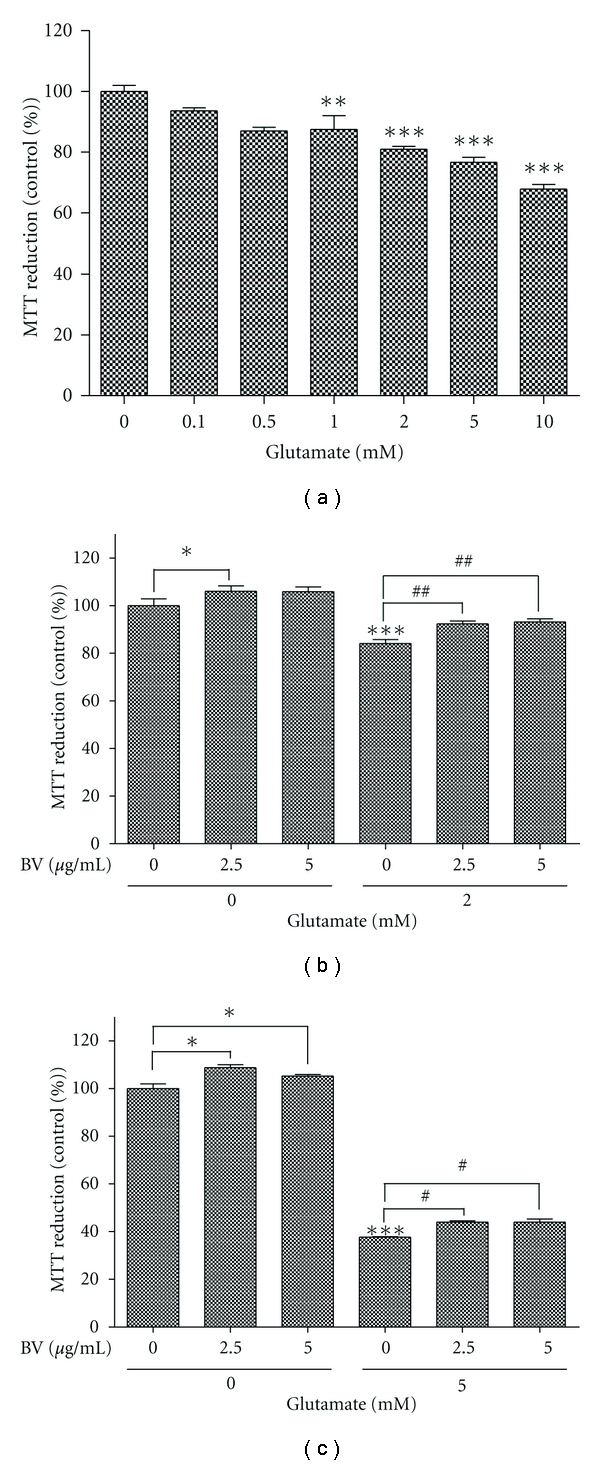
Effect of BV on glutamate-mediated neurotoxicity in N2a neuronal cells. (a) N2a cells were treated with the indicated concentrations of glutamate for 24 h. Cell viability was assessed using an MTT assay. (b) After seeding, N2a cells were treated with the indicated concentrations of BV prior to stimulation with 2 mM glutamate. The effects of BV on glutamate excitotoxicity were evaluated using an MTT assay after 24 h incubation (b) or 72 h incubation (c). The absorbance at 570 nm is expressed as the percent of the relative untreated control cells and is reported as the mean ± S.E.M. of five independent experiments. The values significantly different from the relative controls are indicated with an asterisk. ∗, ∗∗, and ∗∗∗ indicate *P* < 0.05,* P* < 0.005, and* P* < 0.001, respectively, compared to untreated cells. # and ## indicate *P* < 0.05 and *P* < 0.005, respectively, compared to the corresponding untreated control.

To determine whether glutamate induces neuronal cell toxicity, we investigated cell viability following treatment with different glutamate concentrations (Figure 1(a)). For this experiment, N2a cells were seeded in 96-well plates and treated with glutamate (0.1 to 10 mM) for 24 h. At low glutamate concentrations (0.1 to 0.5 mM), glutamate-induced cell toxicity was not observed; however, cells treated with high concentrations of glutamate (1 to 10 mM) showed a significant, glutamate concentration-dependent decrease in viability (Figure 1(a)).

In a previous study, we reported that BV-stimulated cell survival signaling pathways including the ERK and AKT pathways and reduced neuronal cell death [19]. To investigate whether BV pretreatment protects neuronal cell from glutamate-induced excitoxicity, N2a neuroblastoma cells were pretreated with BV (2.5 or 5.0 *μ*g/mL) for 1 h before stimulation with 2 mM glutamate. As shown in Figure 1(b), compared with untreated cells, the glutamate-induced cell toxicity was significantly reduced in the presence of BV (Figure 1(b)). To investigate whether the neuroprotective effect of BV pretreatment is dependent on glutamate treatment duration, we incubated N2a cells with 5 mM glutamate for 72 h after BV (2.5 or 5.0 *μ*g/mL) pretreatment for 1 h. BV (2.5 or 5.0 *μ*g/mL) pretreatment increased MTT reduction significantly compared to 5 mM glutamate-treated N2a cells (*P* < 0.05, Figure 1(c)). In addition, we observed that BV treatment (2.5 or 5.0 *μ*g/mL) increased cell viability compared to untreated N2a cells (Figures 1(b) and 1(c)). These results suggest that BV treatment at the proper dose could attenuate glutamate-induced neuronal cell excitoxicity.

### 3.2. BV Pretreatment Reduces Glutamate-Induced Microglial Cell Death

**Figure 2:**
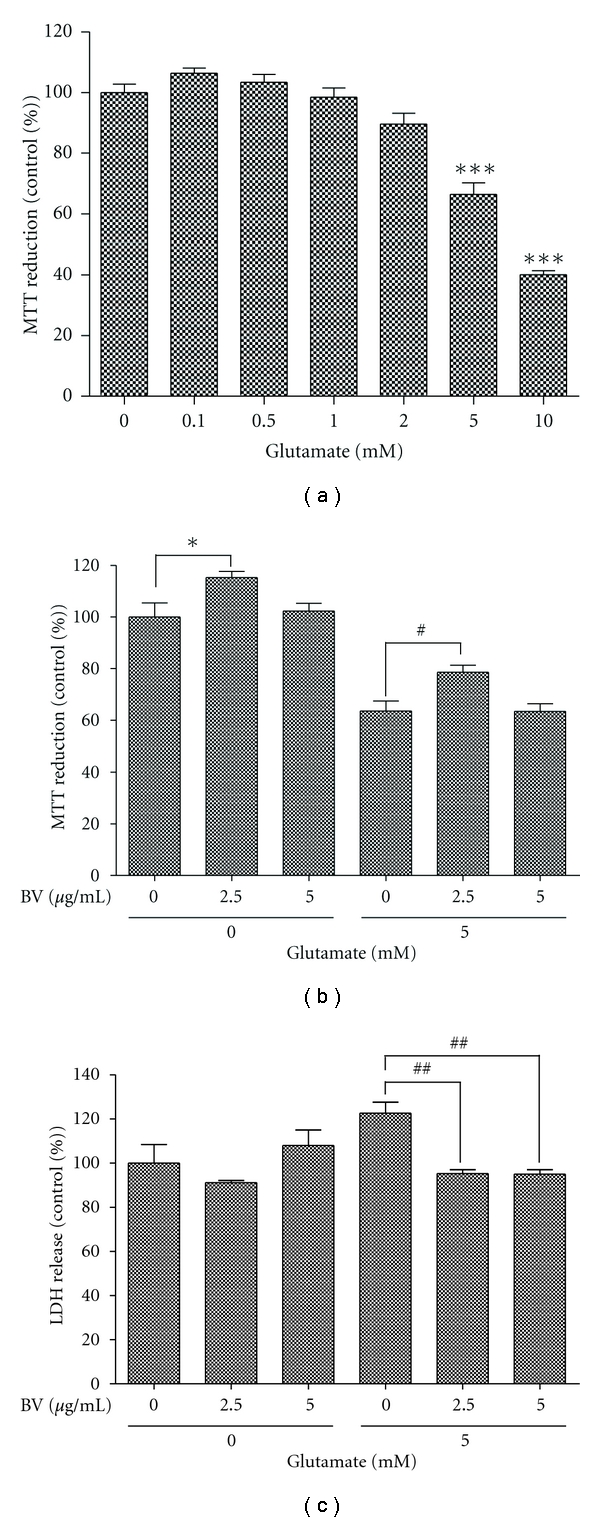
Effect of BV on glutamate-mediated toxicity in BV2 microglial cells. (a) BV2 cells were treated with the indicated concentrations of glutamate. Cell viability was measured using an MTT assay. (b) BV2 cells were pretreated with 2.5 or 5 *μ*g/mL BV for 1 h and then stimulated with 5 mM glutamate or vehicle for 24 h. An MTT assay was then performed to evaluate cell toxicity. (c) LDH release by BV- or vehicle-pretreated BV2 cells stimulated with glutamate for 24 h or vehicle was quantified. All values are expressed as the mean ± S.E.M. from five independent experiments. ∗∗∗ indicates *P* < 0.001 compared to untreated cells, and # and ## indicate *P* < 0.05 and* P* < 0.005, respectively, compared to glutamate-treated BV2 cells (Glu).

Recently, microglia have been linked to glutamate metabolism. In the BV2 microglial cell line, glutamate induces apoptosis and accumulates in the mitochondria, resulting in activation of the caspase-dependent apoptosis cascade [20]. To examine the cell toxicity of glutamate in microglia, BV2 cells were treated with 0.1 to 10 mM glutamate for 24 h, and cell viability was measured using the MTT assay. As expected, glutamate induced concentration-dependent cell toxicity (Figure 2(a)). Next, we pretreated BV2 cells with 2.5 or 5 *μ*g/mL BV to examine the protective effect of BV against glutamate-induced toxicity in microglial cells. As shown in Figure 2(b), while 5 mM glutamate decreased BV2 cell viability to 60% of that measured in control cells, cells pretreated with 2.5 *μ*g/mL BV prior to incubation in 5 mM glutamate showed a 20% increase in viability compared to cells not pretreated with BV. However, pretreatment with 5 *μ*g/mL BV did not protect against glutamate-induced microglial cell death (Figure 2(b)). To confirm this result, we performed a lactate dehydrogenase release (LDH) assay. However, we observed that pretreatment with 2.5 *μ*g/mL or 5.0 *μ*g/mL BV prior to 5 mM glutamate reduced LDH release by approximately ~30% compared to the 5 mM glutamate-treated BV2 cells (Figure 2(c)). Based on these findings, the protective effect of BV could potentially reduce the neuroinflammatory events related to activated microglial cells.

### 3.3. BV Pretreatment Reduces the Glutamate-Stimulated Phosphorylation of JNK, ERK, and p38 and Increases the Expression of Activated AKT in N2a Cells

**Figure 3:**
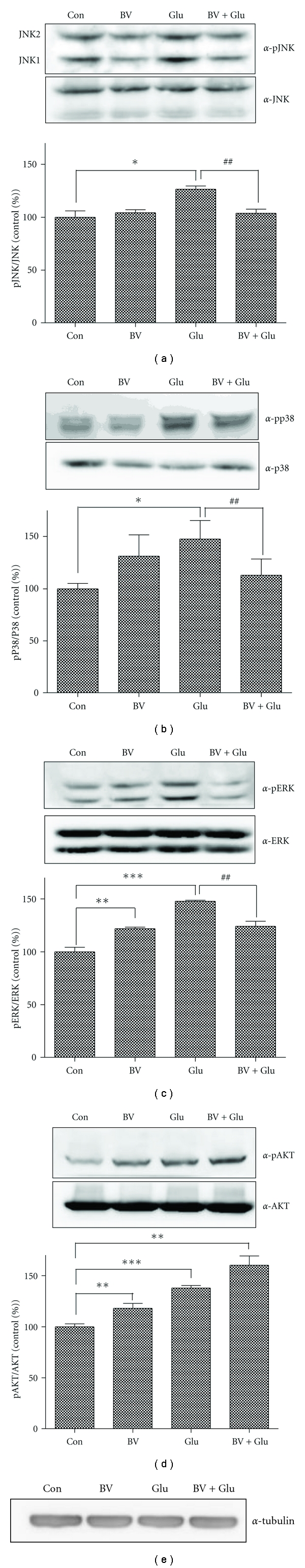
BV suppresses the activation of glutamate-mediated signaling in N2a neuronal cells. To determine the mechanism by which BV protects against glutamate-induced cell toxicity, N2a cells were preincubated with 2.5 *μ*g/mL BV for 30 min prior to treatment with 5 mM glutamate for 30 min. (a) Total cell lysates were separated using 10% SDS-PAGE and western blots were performed using antiphospho JNK or JNK antibodies. (b) Glutamate-induced p38 activation was determined by anti-pp38 antibody. (c) The expression level of phospho-ERK was investigated with anti-pERK antibody. (d) The expression of phospho-Akt was measured with antiphospho-Akt antibody in N2a cells. (e) Levels of tubulin were measured to control for protein loading. Immune blots were quantified with the relative phospho-/nonphospho- ratio as indicated. ∗, ∗∗, and ∗∗∗ indicate *P* < 0.05, *P* < 0.005, and *P* < 0.001, respectively, compared to untreated cells. ## indicates *P* < 0.005 compared to glutamate-treated N2a cells (Glu). Con: control, BV: BV-treated cell, Glu: glutamate-treated cell, and BV+Glu: BV pretreated-cells prior to glutamate treatment.

To assess the mechanism by which BV reduced glutamate-induced toxicity, we investigated the effects of BV on the ERK, JNK, and p38 signaling pathways. Glutamate-treated N2a cells exhibited a 30% increase in activated JNK compared to untreated or BV-treated cells (Figure 3(a)). Interestingly, we observed that BV pretreatment reduced levels of phospho-JNK in N2a cells preincubated with 2.5 *μ*g/mL BV and then treated with 5 mM glutamate (BV+Glu N2a cells) (Figure 3(a)). p38, which is involved in cell death signaling pathways, was also activated by glutamate stimulation; however, the activation of p38 was suppressed in N2a cells preincubated with BV and then treated with glutamate (BV+Glu N2a cells) (Figure 3(b)). ERK signaling has been shown to be important in both cell survival and cell death [21, 22]. In this study, we found a 1.4-fold increase in phospho-ERK levels following glutamate treatment; following BV pretreatment, glutamate-induced phospho-ERK levels were reduced 1.2-fold (Figure 3(c)). The serine/threonine protein kinase Akt/PKB also has a role in multiple cellular processes including cell proliferation, apoptosis, and cell death [23]. As shown in Figure 3(d), BV or glutamate treatment increased Akt phosphorylation at Ser435 1.2- to 1.3-fold compared to untreated N2a cells, and the activation of Akt was 1.5-fold greater in N2a cells preincubated with BV and then treated with glutamate (BV+Glu N2a cells). These results suggest that BV pretreatment suppresses glutamate-mediated activation of cell death signaling (pJNK, pp38, and pERK) and increases PI3K signaling pathways (pAkt).

### 3.4. BV Treatment Suppresses Glutamate-Mediated JNK Activation in BV2 Cells

**Figure 4:**
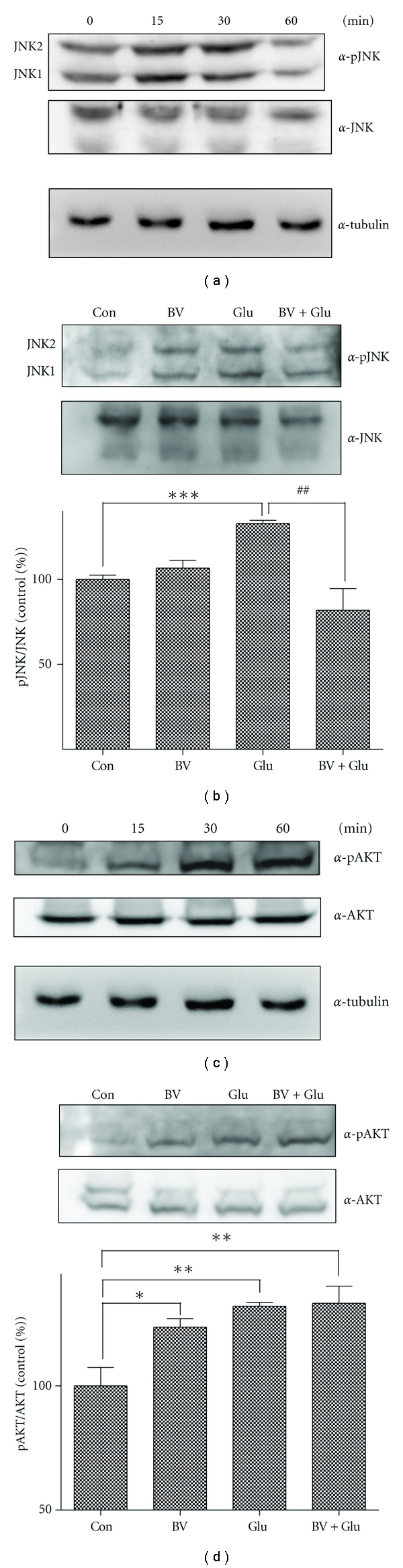
Glutamate-mediated changes in protein expression in BV2 cells and effects of BV pretreatment. BV2 cells were seeded in 60 mm plates (2 × 10^6^/plate). (a) After a 24-h incubation, BV2 cells were treated for the indicated incubation time with 5 mM glutamate. Cell lysates were analyzed with western blotting with phospho-JNK or JNK antibodies. (b) BV2 cells were treated with 2.5 *μ*g/mL BV for 30 min prior to stimulation with 5 mM glutamate for 30 min. (c) After a 24-h incubation, BV2 cells were treated for the indicated incubation time with 5 mM glutamate. Cell lysates were analyzed with western blotting with phospho-Akt or Akt antibodies. The activation of Akt was calculated based on the relative phospho-/nonphosphor- ratio as indicated. (d) BV2 cells were treated with 2.5 *μ*g/mL BV for 30 min prior to stimulation with 5 mM glutamate for 30 min. Western blot analysis was performed with phospho-Akt or total Akt antibodies and evaluated based on activated Akt/Akt ratio. Levels of tubulin were measured to control for protein loading. ∗ indicates *P* < 0.05 compared to untreated cells, and ## indicates *P* < 0.005 compared to the corresponding control.

BV2 cells are an immortalized murine microglial cell line. Glutamate-induced excitotoxicity in microglia can cause neuroinflammation in AD and ALS models [24, 25]. To evaluate the effects of BV on glutamate-induced cell toxicity in BV2 microglia, we investigated whether BV pretreatment affects glutamate excitotoxicity in BV2 cells. As seen in Figure 4(a), we confirmed the activation of JNK in 15–30 min, in accordance with the result seen in BV2 cells (Figure 4(a)). In addition, the glutamate-induced increase in the expression of phospho-JNK was decreased 1.5-fold by pretreatment with 2.5 *μ*g/mL BV (Figure 4(b)). Furthermore, incubation with 5 mM glutamate increased Akt phosphorylation in a time-dependent manner (Figure 4(c)), and pretreatment with 2.5 *μ*g/mL BV increased the expression of phospho-Akt in BV2 cells (Figure 4(d)). Taken together, these results suggest that BV pretreatment could reduce inflammatory cell responses resulting from microglial cell-associated glutamate excitotoxicity.

## 4. Discussion

Recent reports on neurodegenerative diseases have shown that alterations in protein kinase expression and activity can modify the downstream activation of signaling proteins and trigger neuronal loss. Glutamate is the predominant excitatory neurotransmitter in the CNS, and excessive cellular glutamate release has been connected to excitotoxic events in neurodegenerative diseases. In this study, we determined if BV regulates cell viability and protein expression in normal neuronal and microglial cells. We observed dose-dependent glutamate-mediated toxicity in both N2a neuroblastoma and BV2 microglial cells. However, BV pretreatment prior to glutamate stimulation protected the cells from cell death. In the case of the BV2 cells, we observed different results in the LDH and MTT assays for cell viability, which suggests a BV-mediated neuroprotective effect against glutamate (Figures 2(b)-2(c)). Because LDH release is proportional to the rate of pyruvate loss, this parameter may not precisely evaluate cell viability in neuronal cells.

According to previous reports, glutamate can either protect neurons against glutamate-mediated excitotoxicity [26] or induce apoptosis. Key players in the cellular responses to various stimuli include ERK, p38, and JNK [27]. Additionally, the Ras-MAPK pathway is involved in various cellular functions including survival, long-term potentiation, and synaptic plasticity [28]. However, the role of the MAPK pathway in neuronal protection is controversial and depends upon the duration of ERK activation [28]. Several reports have suggested that the MAPK-ERK1/2 signaling pathway promotes cell survival through the activation of CREB [23]. Activation of ERK has also been shown to contribute to neuronal death in some models of neurotoxicity [21, 22]. In this paper, we found that BV inhibited the glutamate-induced phosphorylation of ERK, p38, and JNK in N2a neuroblastoma cells and glutamate-induced JNK phosphorylation in BV2 microglial cells. The optimized concentration of BV (2.5 *μ*g/mL) increased phospho-AKT expression [23]. The activation of MAPKs, including ERK, p38, and JNK [29–32], is promoted by protein kinase C (PKC). Previous studies have demonstrated that ERK is involved in growth-associated responses and that JNK and p38 are activated by cytotoxic stresses, including the cytokine-like lipopolysaccharide (LPS) [29]. However, other reports have shown that BV increased the phosphorylation of JNK in Raw 264.7 macrophages and synoviocytes [33]. We found that, in N2a neuroblastoma cells, AKT levels were upregulated by treatment with BV and glutamate and that BV combined with glutamate stimulation was the most effective at increasing AKT expression in N2a neuroblastoma cells. In addition, we observed higher cell mortality because of glutamate in microglial cells compared to N2a cells. These results suggest that BV can protect against glutamatergic damage. In BV2 microglial cells, pretreatment with BV decreased glutamate-induced JNK upregulation compared to glutamate-treated cells. We hypothesize that BV could be effective against cell stress and that it may contribute to cell survival via decreasing JNK protein levels following exposure to increased glutamate concentrations. In addition, BV pretreatment increased the activation of AKT in BV2 cells. Furthermore, BV combined with glutamate treatment modestly upregulated AKT phosphorylation in BV2 microglial cells. Taken together, our results suggest that BV-induced AKT activation contributes to cell survival in both BV2 microglial cells and N2a neuronal cells. 

Amyotrophic lateral sclerosis (ALS) is a fatal paralytic disorder characterized by the selective death of motor neurons. Approximately 10% of ALS cases are inherited (FALS) and 90% are sporadic (SALS). About 25% of the FALS cases are caused by missense mutations in the ubiquitously expressed enzyme Cu^2+^/Zn^2+^ superoxide dismutase (SOD1). Several reports support a role for oxidative stress in neuronal death during aging and in neurodegenerative diseases (e.g., ALS). In addition to reactive oxygen species, glutamate may also be involved in ALS [34]. Although paralysis in ALS results from the death of motor neurons, the cellular autonomy of ALS pathogenesis has been questioned by studies in which mutant SOD1 was selectively deleted in microglia and astrocytes; these studies emphasize the contribution of these cells to ALS pathogenesis.

To the best of our knowledge, this study is the first to show that BV inhibits cell death and activation of proapoptotic signaling in glutamate-stimulated cells. We also show that BV attenuates cell toxicity though inhibition of the JNK and p38 pathways. These findings emphasize the clinical importance of BV for the treatment of glutamate-mediated syndromes and inflammatory diseases. Further investigation of this activity *in vivo *is necessary to elaborate the mechanisms involved and to permit the full exploitation of the therapeutic potential of BV. To test the effectiveness of BV in treating ALS, future studies are needed to elucidate the signaling responses activated by glutamate or BV in neurons from mutant hSOD1 transgenic mice. In addition, BV contains a variety of peptides (e.g., melittin and apamin), enzymes (e.g., PLA2, histamine, and epinephrine), nonpeptide components including lipids and carbohydrates, and free amino acids. Therefore, further research is required to determine bioactive single element of BV.
